# Global Food Security under COVID-19: Comparison and Enlightenment of Policy Responses in Different Countries

**DOI:** 10.3390/foods10112850

**Published:** 2021-11-18

**Authors:** Xiaoyu Jiang, Yangfen Chen, Jieyong Wang

**Affiliations:** 1 Institute of Agricultural Economics and Development, Chinese Academy of Agricultural Sciences, Beijing 100081, China; jiangxiaoyu0022@126.com; 2 Institute of Geographic Sciences and Natural Resources Research, Chinese Academy of Sciences, Beijing 100101, China

**Keywords:** global food security, COVID-19, policy responses, risk management

## Abstract

(1) Background: COVID-19 has exacerbated global food security risks, and the global food supply chain, especially in developing countries, has become more vulnerable. (2) Methods: In this paper, we discussed the current security of global food, response measures, and potential impacts, and analyzed the characteristics and evolution of food security policies in four representative countries: China, Italy, Malawi, Argentina. (3) Results: The results showed that most countries give priority to ensuring food access. Most underdeveloped countries adopt humanitarian intervention measures such as food distribution and transfer payments, while developed countries tend to implement development intervention policies such as supporting small- and medium-sized enterprises and guaranteeing employment. (4) Conclusions: Despite the ample global supply, developing countries still face long-term food security risks, highlighting the importance of strengthening global food security governance and risk management. Finally, a food security risk response policy framework was built to provide suggestions for effectively handling COVID-19 and similar public health emergencies in the future.

## 1. Introduction

The COVID-19 pandemic has become a public health emergency of global concern. It brings not only a health crisis, but also a series of social, economic, and food security issues [1]. Farmers, families, to countries, and even the world has been affected [2]. The Global Report on Food Crises shows that COVID-19 exacerbated pre-existing fragilities [3]. According to The State of Food Security and Nutrition in the World 2021 jointly released by FAO and other international organizations, around 660 million people may still face hunger in 2030, in part due to lasting effects of the COVID-19 pandemic on global food security—30 million more people than in a scenario in which the pandemic had not occurred [4]. Although FAO predicts that the global food supply will be sufficient in 2020 [5], too many people are still hungry, highlighting the fragility of the current food system [6,7]. From the perspective of the four dimensions of food security (availability, access, utilization, and stability), developing countries still face the challenge of ensuring food access, while how to maintain the stability of the global food system is an urgent problem that needs to be solved for developed countries. How to ensure food security while responding to the pandemic, and how to effectively deal with similar public emergencies in the future are important issues that all countries need to consider.

The crisis brought about by COVID-19 is not only an inspection of the food security of all countries, but also an important test of global food security governance. Many scholars have emphasized the challenges of food security, such as food and water safety, supply chain and trade disruption, consumer food behavior, diet and nutrition, food surveillance and technologies, and discussed the impact of policies such as raising import tariffs and increasing agricultural inputs on food security [8,9]. The current research subjects are mostly for single countries or regions, and there is a lack of comparative analysis of global food security policies. The research content focuses on the adverse effects of COVID-19 on food security, but an effective and rapid response policy framework has not yet been proposed to deal with future food security challenges. In order to fill the research gap, we put forward the following research questions: From the beginning of the pandemic to now, what new characteristics and trends have been shown in the response measures? For countries with different levels of economic, social, and agricultural development, which aspects are their response measures focusing on? What are the effects of these policies and what are the implications for responding to similar public emergencies in the future?

The novelty of this article is embodied in two aspects. At the practical level, countries with different levels of development may have differences in their coping capabilities and policy goals. This paper categorizes countries in terms of economic strength, agricultural development level, and region, and systematically sorts out the orientation, similarities, and differences in food policies of different types of countries in response to COVID-19. Compared with the study of a specific single country, this paper presents a panoramic display of the food security policies in response to COVID-19 from a global level, which is helpful for the learning and accumulation of experiences among countries. At the theoretical level, the analysis of food security policy mostly considers the aspects of availability, access, utilization, and stability. This article not only analyzes the impact of different countries’ response policies on the four dimensions of food security, but also considers risk factors. The policy measures introduced by different countries are actually responses to the systemic risks caused by COVID-19 in the food supply chain. Therefore, we try to propose a policy framework for future responses to similar public health emergencies from a risk management perspective.

In addition to the introduction, we first reviewed the relevant research progress, and introduced the research methods and data. Then, we analyzed the global food security situation, countries’ response measures and possible policy implications under COVID-19, and summarized the regular pattern of policy evolution in different types of countries. Four representative countries, China, Italy, Malawi, and Argentina, were used as case studies to explore their policy characteristics in the field of food security. Finally, we have established a food security risk response framework under the pandemic from the perspective of risk management, hoping to provide a systematic solution to the food security crisis caused by similar public health emergencies.

## 2. Literature Review, Materials and Methods

### 2.1. Literature Review

In order to alleviate the food security crisis brought about by the pandemic, reactive and proactive solutions are necessary [10]. In the short term, existing relationships and short-term supply chains have been rapidly utilized [11]. In terms of social security, policies that support disadvantaged families and ensure adequate employment opportunities were adopted by most countries [12,13]. In terms of agricultural production support, some countries have implemented seed safety interventions [14]. In terms of food distribution, food purchase programs can deliver agricultural products to consumers, thereby minimizing the health and economic crises of the most vulnerable people [15].

The blockade is a controversial response. Although it is effective in controlling the pandemic, it not only has high economic costs, but also brings a series of challenges to food acquisition, supply, distribution and transportation [16,17]. For urban areas, social distancing measures reduce jobs and incomes, leaving the poorest people unable to pay rent and buy food [18]. The closure of the market caused a sharp increase in food prices [19], which particularly affected the urban poor, further highlighting inequality [20]. Long-term school closures prevent most students in low- and middle-income countries from accessing meals, which may reduce student nutritional intake and family food safety [21,22,23]. The negative and unequal effects of the blockade on the economy and food security may mean that it is unsustainable in the long run [24].

Meanwhile, short-term trade protectionist measures have also adversely affected the food security of import-dependent countries and disrupted the global supply chain [25]. Although international organizations, governments and trade economists have called for avoiding trade protectionist measures to prevent food prices from rising, some countries have issued bans on agricultural exports. The combined effect of border closures and movement restrictions increases food losses and export costs [26].

In the long term, the lessons learned from the COVID-19 crisis may drive the development of new sustainable agricultural policies [20]. Researchers generally believe that a resilient food system is necessary [27]. The problem of pandemic response needs to be resolved through the formulation of policies tailored to local conditions and should be implemented in a humane manner [28]. The management, decision-making, communication, implementation of COVID-19 and the review of the effectiveness of new and existing measures require careful organization and control [29].

At present, these actions are still inadequate, with time lag and insufficient coordination to contain the food and nutritional insecurity crisis [30,31]. When designing policies and interventions, countries should not only be aware of the seriousness of the situation, but also strengthen or relax measures at the appropriate time according to the spread of the pandemic [32]. Protecting the food supply chain, avoiding export bans, and rationally using food storage should be wise choices for countries [33,34].

Overall, existing research helps to understand the impact of current response measures on food security. However, most of researchers use inquiry or online survey methods, and target one or several specific countries. There are few systematic research and regular summaries of the response measures of different types of countries. This is essential for countries to find their positions and learn from the experience of other countries to effectively respond to the impact of the pandemic on food security.

### 2.2. Methodology

The argumentation of the main conclusions of the article is based on the application of comparison, statistics, graphs, and table analysis methods. The comparative analysis method and case study method were used to carry out this research. These methods can be used in public policy analysis [35,36,37]. Comparative policy research has made a great contribution to clarifying the nature of policy analysis and policy formulation processes that have led to the construction of these policy portfolios. It studies how to construct an effective combination of policy tools to effectively achieve complex policy goals, and through a more specific and traditional focus upon what constitutes the effectiveness of particular types of policy tools within a mix [38]. We used a combination of horizontal comparison and vertical comparison to achieve an in-depth understanding of food security policies. Horizontal comparison is to compare related things in the same period, while vertical comparison is to compare the specific characteristics of the same object in different periods. On the basis of classifying policies as producer oriented, consumer oriented and trade oriented, we horizontally compared the characteristics of different regions (Africa, North America, Asia and the Pacific, Europe, Latin America and the Caribbean, Near East) and countries with different levels of economic development, which can understand the similarities and differences of policies in different regions in response to C0VID-19, and then analyzed the reasons for this difference. According to World Bank standards, countries are divided into low-income economies, lower-middle-income economies, upper-middle-income economies and high-income economies. In case analysis, we applied vertical comparison to reveal the policy characteristics and development trends of specific countries in different periods and stages.

In order to get more detailed results, we also conducted a case study. Case studies can bring more in-depth knowledge, especially complex social issues involving many participants [39]. In this study, it provides background information on the introduction of food security policies in a specific country, as well as important knowledge and insights on motivation, resources, support, and time. Four typical countries, China, Italy, Malawi, and Argentina, were selected to analyze their policies in response to COVID-19 (Table 1). When selecting a specific country as a case study, four factors should be considered—economic development level, agricultural resources, agricultural productivity and food import and export—because they are closely related to the four dimensions of food security (availability, access, utilization, and stability). We select GDP per capita, per capita arable land, grain yield, and import and export indicators to represent these four key considerations. GDP per capita is an important indicator to measure economic level of a country, and it is related to the country’s ability to allocate resources. Countries with lower economic levels may not be able to use more funds to deal with COVID-19, such as food distribution and transfer payments, which will directly affect the access and utilization of food. Per capita arable land and grain yield can respectively represent agricultural resource endowment and productivity level of a country. Countries with insufficient arable land and low levels of agricultural productivity may encounter problems such as rising food prices and shortages of food supplies under the impact of the epidemic. Food import and export indicators can reflect the role of a country in the global food supply chain. The policies of food exporting countries will have an important impact on global food supply. For countries that rely on imported food, how to ensure stable domestic food supply under COVID-19 will be an important challenge.

As a country with an early outbreak of COVID-19, China is also the world’s largest food importer. Its response measures are unique and of high research significance. Italy has a high level of economic development and is also one of the European countries most severely affected by COVID-19. It is meaningful to study its food security policy as a representative of developed countries. Malawi is located in the least developed sub-Saharan Africa, with low agricultural technology and per capita food availability. As an important food producer and exporter in the world, Argentina’s response measures are not only of great significance for ensuring domestic food security, but also have a certain impact on global food supply. Although these four countries cannot represent all countries, they can roughly reflect the basic situation and policy characteristics of similar countries, taking into account the guiding role of the government’s relevant response measures such as economic development level, resource endowment, agricultural productivity, imports and exports.

### 2.3. Data Sources

The Food and Agricultural Policy Decision Analysis database (FAPDA) established by FAO was used to analyze the characteristics of food security policies. The FAPDA database was established during the global food price crisis in 2008, when it was used to collect food security decision-making information. After the outbreak, FAO expanded the database and added a new column to collect official decisions to reduce the impact of the pandemic on the food and agricultural systems. On this platform, countries can share their initiatives and search for practices in other countries. Due to different national conditions, in the face of the pandemic, the policies adopted to strengthen the food system are also different. FAO believes that best practices in response to the pandemic come from the experience of different countries, which can help other countries make more informed decisions and predict future challenges.

It provides a variety of search methods: search by country, time, and commodity; search by target, such as consumers, producers, or trades; or search by subject, such as nutrition, taxation, or natural resource management. In this paper, the keywords and logic (producer oriented, consumer oriented, and trade oriented) we extracted during policy analysis come from the way we search by target. The time and legal status of policies are also marked. The database is continuously updated, and a simple and clear new policy action submission module has been designed for users. The submitted content will be added to the database after verification by the FAPDA working group. Currently, the database has been opened to all member states. There are six topics under the platform: emergency response, nutrition, trade, social security, development and transformation, as well as incentives and restraint measures to help countries make wise choices and help accelerate the establishment of an inclusive overall policy framework.

Compared with developed countries, the food security situation affected by COVID-19 in developing countries deserves more attention. In order to make up for the limitations of the FAPDA database and have a thorough understanding of the actual impact of COVID-19 on certain countries and the effects of these countries’ policies, the Macro Poverty Outlook (MPO) report and High-Frequency Phone Surveys data of World Bank were introduced in case analysis. The MPO is released twice annually for the Spring and Annual Meetings of the World Bank Group and International Monetary Fund. It consists of individual country notes that provide an overview of recent developments, forecasts of major macroeconomic variables and poverty during 2020–2022, and a discussion of critical challenges for economic growth, macroeconomic stability, and poverty reduction moving forward. The High-Frequency Phone Surveys data of World Bank was used to supplement how COVID-19 affects household food security and policy responses in Malawi. Malawi High-Frequency Phone Survey COVID-19 (HFPS COVID-19) was implemented by the National Statistical Office (NSO) on a monthly basis during the period of May 2020 and June 2021. The households were drawn from the sample of households interviewed in 2019 as part of the Integrated Household Panel Survey (IHPS 2019). These data contribute to filling critical gaps in information that could be used by the Malawian government and stakeholders to help design policies to mitigate the negative impacts.

## 3. Results

### 3.1. Global Food Security under COVID-19

COVID-19 has exacerbated global food insecurity and has attracted the attention of international organizations such as the FAO, WHO, and IMF. Most countries have adopted countermeasures. In this section, we will discuss the global food security situation under COVID-19, the policy responses of different countries, and the possible effects of the policies.

#### 3.1.1. Global Food Security Situation

COVID-19 threatens the four dimensions of food security, and its impact is profound and lasting. In the short term, COVID-19 directly affects people’s food access. To control the pandemic, markets and restaurants were closed, school dining plans were disrupted, and people had limited access to food [20]. In addition, unemployment and income reduction caused by the pandemic, as well as increased food prices in some countries with insufficient food supply, also reduced people’s purchasing power and indirectly affected food access [40]. In the medium term, COVID-19 will affect the availability and utilization of food. Transport regulation has restricted the movement of agricultural labor, which is not conducive to food production [41]. Agricultural production is affected [14,42]. For consumers, especially people in developing countries, declining income and insufficient supply of high-value-added agricultural products will change their consumption habits, reduce their intake of vegetables, meat, eggs, and milk, and force them to buy cheap and low-nutrition foods [43]. This reduces dietary diversity and micronutrient intake, which is not conducive to people’s nutrition and health [44]. In the long run, COVID-19 will also threaten the stability of the food system. The pandemic has intensified the volatility of food prices and caused uncertainty in food supply and markets, thereby reducing domestic and international agricultural investment, which is of great significance for improving productivity and food quality [44].

**Figure 1:**
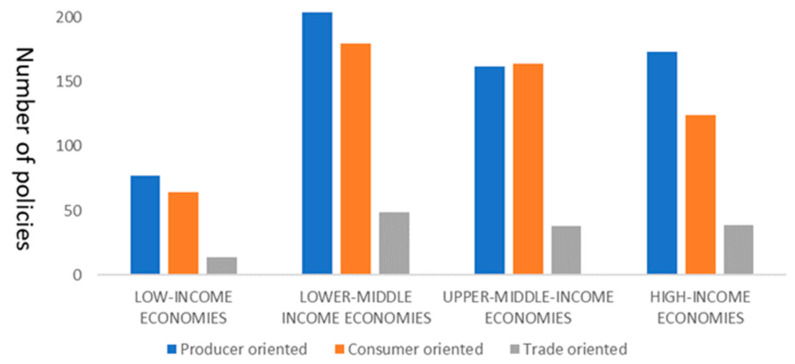
Characteristics of food security policies in four types of countries in response to COVID-19.

The global economic recession and disruption of food supply chains caused by COVID-19 are threatening livelihoods and food security, exacerbating risks for countries already facing food crises and the most vulnerable people working in the informal sector. Democratic Republic of the Congo is now facing the most serious food security crisis, with 21.8 million people in a state of extreme food insecurity. Burkina Faso, Nigeria, Somalia, and Sudan also experienced severe hunger. The FAO estimates that 45 countries (34 countries in Africa) need external food assistance [45]. We have studied the characteristics of measures issued by countries with different levels of economic development in response to COVID-19 from producer oriented, consumer oriented, and trade oriented perspectives. According to the World Bank’s latest country classification (2020–2021) by income level, countries can be divided into high-income economies, upper-middle-income economies, lower-middle-income economies, and low-income economies. From Figure 1, the food security policies of the four types of countries are concentrated on the producer- and consumer-oriented. In terms of the number of policies, lower-middle-income countries attached the greatest importance to addressing food security risks. In contrast, low-income economies faced the greatest food security risks, but have not received enough attention. In addition, low-income economies faced serious risks of hunger before the outbreak of COVID-19, such as armed conflicts, prolonged economic recession, heavy rains and floods, and their food security situation will be even worse.

Note: Relevant data is calculated by authors based on the FAPDA database of FAO, and the data is as of 31 December 2020.

#### 3.1.2. Policy Responses of Different Countries

**Figure 2:**
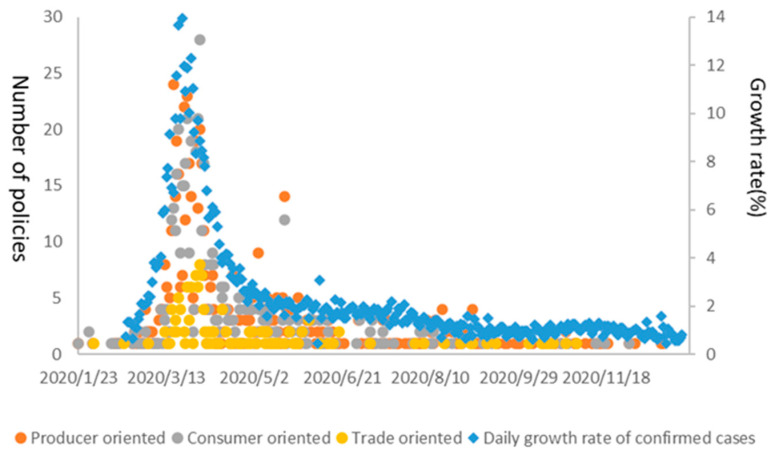
Number of food security policies in response to COVID-19.

Since January 2020, countries have implemented a series of food security policies. As shown in Figure 2, the time for countries to introduce food security policies coincides with the spread of the pandemic. On 23 January 2020, China implemented measures such as locking down the city, reducing value-added tax for small taxpayers in Hubei Province, and requiring National Farmers’ Cooperatives to participate in the control of COVID-19. In February, some developed countries, such as Singapore, South Korea, and Italy, stepped up their efforts to fight the pandemic in terms of transport regulation, increased agricultural budget expenditures, reductions or delayed payment of taxes. Subsequently, the pandemic spread to Latin America, Africa, and other places. The growth rate of daily confirmed cases in mid-to-late March exceeded 10%. The number of food security measures also reached its peak. The measures mainly included transport regulation, food distribution, reduction of import tariffs, promotion of employment, and transfer payments to small- and medium-sized enterprises and vulnerable people. During this period, the state’s intervention was the greatest, and the policy emphasized fairness and protection of vulnerable people. After June, the number of new policies gradually decreased, and the policy focus gradually shifted from controlling the pandemic and ensuring food supply to promoting employment and economic development.

Note: Relevant data are calculated by authors based on the WHO Coronavirus (COVID-19) Dashboard and the FAPDA database of FAO.

From a regional perspective, as shown in Table 2, countries generally attach importance to supporting small- and medium-sized enterprises and protecting vulnerable people, such as transfer payments, food distribution, credit support, etc., focusing on maintaining employment and purchasing power to ensure food access. Blockade and quarantine measures are effective means to prevent the spread of COVID-19, but many countries have eliminated restrictions on the transportation of agricultural products. Due to differences in medical and health conditions, agricultural technology, and economic development, the focus of policies varies from country to country. The measures adopted by less developed countries mainly include food distribution, transfer payments, subsidies, and price control, while developed countries mainly introduced economic policies such as financial support, credit support, and employment plans. Countries that rely on imports of food implemented export restrictions or bans to ensure domestic basic food supplies. In general, the policies of less developed countries focused on responding to the adverse effects of the pandemic on food security and ensuring that people could get enough food in the short term. In addition to conventional countermeasures, developed countries tended to implement incentive measures to replace direct intervention, and were more concerned with how to develop the social economy impacted by COVID-19 in the long term.

#### 3.1.3. Possible Effects of the Policies

The government’s active response will alleviate the potential food security crisis brought about by COVID-19. At the level of food availability, when transportation was blocked during the pandemic, many countries took measures to deliver production inputs to farmers, which guaranteed the progress of agricultural production to a certain degree [46]. In addition, the world’s major food producing and exporting countries have a warm climate, and the harvest prospects of major crops are promising. At present, the impact of COVID-19 on the global food supply is limited. At the level of food access, humanitarian interventions provided to low-income people, temporary workers and families in need help ensure their purchasing power and improve their nutrition and health [47]. Ensuring that supermarkets or enterprises that sell food are operating, conducting sales services, and intervening in food prices will also restrain the impact of panic consumption on food prices [48]. At the level of food utilization, the scarcity of food during the pandemic and changes in cooking can help reduce food waste [49]. The food safety measures promulgated by many countries are essential to ensure food cleanliness and human health, and are conducive to the full utilization of food [50]. At the level of food stability, at the beginning of the outbreak, major exporters banned or restricted the export of staple foods, and global trade shrunk sharply [51]. However, most restrictive policies have now been cancelled. Most of the countries that are still implementing such restrictions are countries with a small share of the food market, so they have little impact on global food prices and supplies.

### 3.2. Analysis of Food Security Policies in Typical Countries

#### 3.2.1. Food Security Response Measures under COVID-19 in China

**Figure 3:**
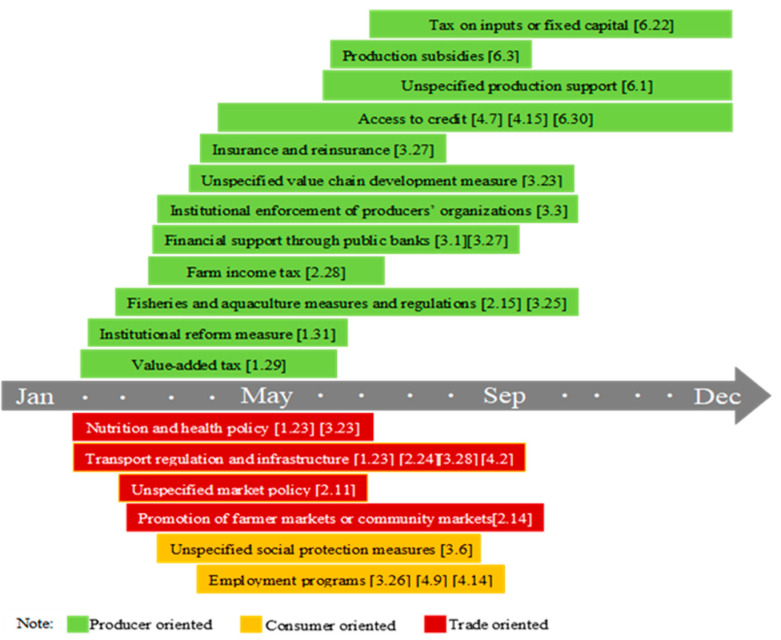
The evolution of food security policy under COVID-19 in China.

China is the world’s largest food importer. The per capita arable land area is only 0.09 hectares, but it has always insisted on self-sufficiency in grains, with a grain self-sufficiency rate of over 90%. As shown in Figure 3, after the lockdown of Wuhan and other cities in Hubei on 23 January 2020, the government’s work focused on strengthening the supply of agricultural products, opening up marketing channels, and strict management of pandemic prevention. From the mid-February, the policy focus shifted to promoting agricultural production. The government provided subsidies to farmers and processing companies, and promoted the restoration of early rice areas. Meanwhile, agricultural enterprises were promoted to speed up the resumption of work and production, paying attention to the transportation and marketing of agricultural materials, and ensuring the needs of spring farming. In addition, the Ministry of Agriculture and Rural Affairs has signed cooperation agreements with the Agricultural Bank of China and China United Insurance Group to promote financial services in rural areas and carry out agricultural insurance. After lifting the blockade of Wuhan on 8 April, the government’s focus shifted to restarting the economy. The government launched a social assistance program to promote the employment of returning migrant workers. At present, the pandemic in China has been controlled, and production and life have been basically restored. This is largely due to the coordination of government humanitarian intervention measures and development intervention measures. At the level of food availability, the planting area of major food crops is basically stable, and the stocks of rice and wheat are sufficient. The foundation for maintaining the overall stability of the agricultural product market is good, and there is no major food crisis. At the level of food access, although the government has adopted strict quarantine policies, necessary food is collectively purchased and delivered to each family, and special channels for agricultural products have been implemented. This can help stabilize the market and reduce panic, which can be regarded as a good experience [40].

Note: The figure shows the food security policy implemented throughout 2020. The data source is the FAPDA database of FAO. “[]” refers to the initial date of the policy.

According to the Macro Poverty Outlook released by the World Bank, economic activity in China has normalized faster than expected, aided by an effective pandemic-control strategy, strong policy support, and resilient exports. Meanwhile, labor markets conditions have improved, and employment has returned to pre-COVID levels. Despite the COVID-19 shock, the government announced in November 2020 that it reached its goal of elimination of extreme (rural) poverty, as measured using the official 2010 poverty standard (equivalent to $2.3/day per person, 2011 PPP). For 2021, poverty is expected to decline to 14.4 percent, representing 32 million fewer poor people than in 2020 [52].

#### 3.2.2. Food Security Response Measures under COVID-19 in Italy

**Figure 4:**
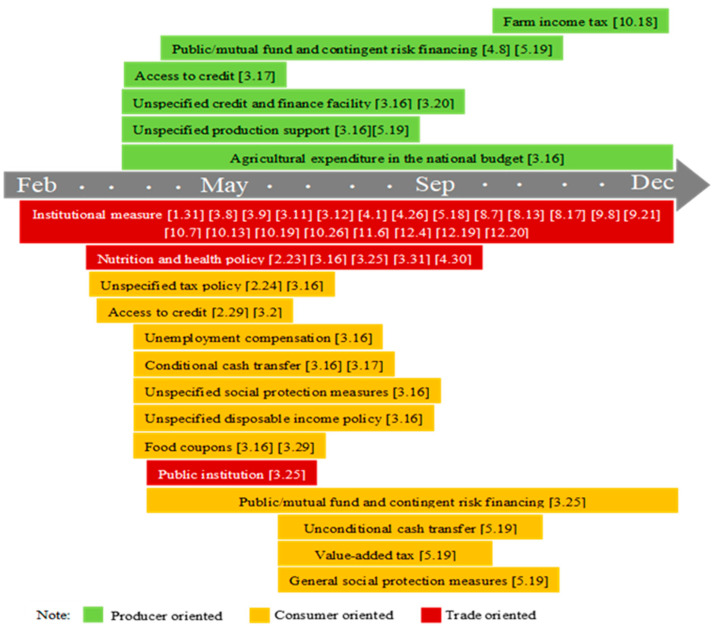
The evolution of food security policy under COVID-19 in Italy.

Italy is a developed industrial country with a per capita GDP far exceeding the world average. As one of the European countries most severely affected by COVID-19, the Italian government issued the Declaration of State of Emergency on 31 January 2020, as shown in Figure 4. Since 9 March 2020, quarantine has been implemented nationwide, and strict travel restrictions have been implemented from 22 March 2020. The government attached great importance to agricultural production and established a 100 million euro fund to support agriculture and fisheries. It paid more attention to social protection, policy measures including the credit for consumption, unemployment compensation, tax payment suspension, cash transfer, paid leave, food assistance fund, food coupons, etc. In addition, since the number of small- and medium-sized enterprises in Italy accounts for more than 98% of the total number of enterprises, they contribute to a large number of jobs and GDP. Enhancing support for small- and medium-sized enterprises is very important for Italy’s economic and social stability. Therefore, the Italian government used a lot of resources to restart the economy, such as providing credit support for small- and medium-sized enterprises, and a large amount of liquidity. In general, at the level of food availability, the government has implemented fewer policies directly related to agricultural production, and the producer oriented policies are mainly targeted to small- and medium-sized enterprises. This is because Italy’s high agricultural productivity and sufficient stocks can guarantee domestic food supply. Additionally, compared to food production, the impact of COVID-19 on food access is more direct and serious [44]. Therefore, the Italian government’s consumer oriented policies are more specific and cover a wide range, involving small- and medium-sized enterprises, low-income groups, the elderly, and children, which are of great significance for ensuring people’s income and employment, improving purchasing power, and promoting food access.

Note: The figure shows the food security policy implemented throughout 2020. The data source is the FAPDA database of FAO. “[]” refers to the initial date of the policy.

#### 3.2.3. Food Security Response Measures under COVID-19 in Malawi

**Figure 5:**
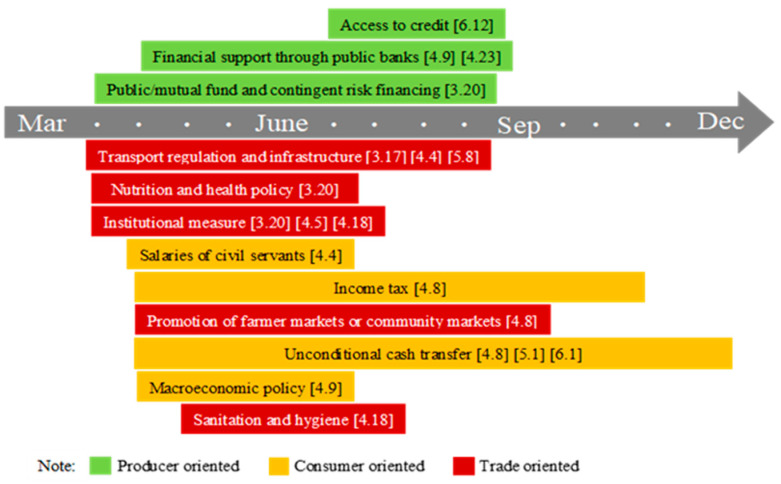
The evolution of food security policy under COVID-19 in Malawi.

Malawi is an agricultural country; 86% of the population is engaged in agriculture. It is one of the least developed countries designated by the United Nations, and its economic development is heavily dependent on foreign aid. Malawi has long faced multiple factors of food insecurity, such as economic crisis, instability and insecurity, extreme weather, plant diseases and insect pests, and animal diseases. Therefore, before the outbreak, the proportion of people with food insecurity and severe hunger in the country was already high [53]. Since mid-March 2020, Malawi has restricted the movement of people, suspended international conferences, closed schools, and other public places (Figure 5). The blockade was implemented nationwide from 18 April to 9 May 2020. The government implemented a US$20 million response plan, and used a lot of resources to recruit medical staff, purchase medical equipment, establish COVID-19 testing centers, even lower the salaries of government staff, and use remaining resources to control COVID-19. In terms of social protection, transfer payments were implemented for low-income people and credit support was provided to small- and medium-sized enterprises.

Note: The figure shows the food security policy implemented throughout 2020. The data source is the FAPDA database of FAO. “[]” refers to the initial date of the policy.

From the perspective of food availability, Malawi’s seasonal rainfall in 2020 was normal, so crop harvest prospects were higher than average. The output of rice, millet and beans is expected to be 8% to 11% higher than 2019. It is expected that poor households in most rural areas will be able to obtain self-produced food, but the opportunity to earn income from crop sales is reduced. From the perspective of food access, the food security of urban low-income households is worse. In Malawi, most people live on less than US$1 a day and cannot afford to purchase necessities during the 21-day lockdown [54]. Although the government has taken some measures to increase people’s disposable income, the situation may not get better soon. Transfer payments and food distribution are temporary measures. With the spread of COVID-19 in the southern regions of the world, the benefits of such humanitarian interventions may not be able to solve the food security problems caused by the deteriorating situation [55]. At the national level, the Macro Poverty Outlook reveals that in 2020, Malawi’s per capita GDP contracted by more than 2%, and the fiscal year’s fiscal deficit expanded to 9.4% of GDP. The share of the population below the international $1.90 poverty line is projected to continue to stagnate around 68 percent in 2021 [52]. At the household level, combined with the data from the High-Frequency Phone Survey on COVID-19 in Malawi (Table 3), the government’s response measures have indeed achieved a certain effect, and the impact of COVID-19 on food security and household income has been reduced. But as of June 2021, 40% of families still worry about not having enough food to eat, and 66.1% of people believe that COVID-19 is a substantial threat to family income.

#### 3.2.4. Food Security Response Measures under COVID-19 in Argentina

**Figure 6:**
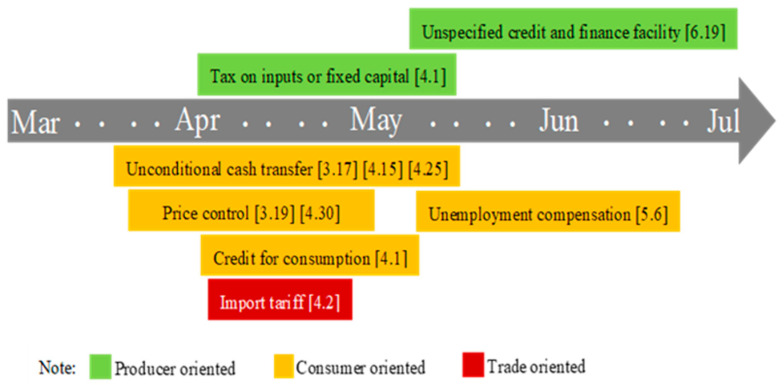
The evolution of food security policy under COVID-19 in Argentina.

Argentina is the third largest economy in Latin America and one of the world’s main producers and exporters of food and meat. Its per capita arable land is among the highest in the world. Argentina’s grain exports account for more than half of their domestic output. The fertile soil and mild climate are suitable for the development of agriculture and animal husbandry, and there are no major problems with domestic food production and supply. However, due to increased domestic concerns about the impact of dry weather on the growth of crops in major producing areas, the price of wheat grains in Argentina rose in August, which intensified seasonal pressure. Meanwhile, stimulated by the sharp depreciation of the country’s currency, the export of agricultural products increased sharply, prompting a rise in domestic prices. With sufficient food supplies, Argentina should devote more energy to ensuring food access (Figure 6). For example, the transfer payments benefited 560,000 retirees and 9 million low-income workers, compensated supermarkets, and local stores to ensure that basic food prices do not rise, provided credit support to consumers, and increased unemployment compensation to US$86 to US$143. There are a few producer oriented support policies, mainly aid programs, which provide smallholder farmers with direct financial support, with a total budget of 420,000 US dollars. As a major food producer and exporter, Argentina has not restricted the export of agricultural products. Regarding trade, it has only reduced the import tariffs on medical and health products. While controlling the pandemic, it also helps ensure the stability of domestic and international food systems. According to the report of Macro Poverty Outlook, with the gradual lifting of the blockade, Argentina’s economic recovery began in the fourth quarter of 2020. Agriculture has almost reached or exceeded the level before COVID. However, the negative impact of the crisis on the performance of the labor market is even more pronounced. More than 20,000 companies have closed in 2020, and 1.4 million people are currently unemployed. In 2021, it is projected that 15.8 percent of the population will be considered poor under the international poverty line of $5.5 per day. A stronger labor market performance is needed to reverse recent poverty increases [52].

Note: The figure shows the food security policy implemented throughout 2020. The data source is the FAPDA database of FAO. “[]” refers to the initial date of the policy.

## 4. Discussion and Policy Implications

### 4.1. Discussion

COVID-19 affects food security from the four dimensions of availability, access, utilization, and stability. Reducing the stress posed by COVID-19 will require collaborative efforts and systemic thinking by stakeholders across all quarters. In the short term, governments have played an important role in ensuring food access during the blockade and isolation period. Through the implementation of key policies, some scholars stress the need for critical agricultural inputs, such as fertilizers and safe, quality seeds, to meet seasonal crop calendars [14]. The effects of policies such as transfer payments and food distribution are worthy of recognition [12,13]. However, in the long run, countermeasures such as blockades and export bans are not sustainable. Many scholars emphasized that the restrictive trade system has compromised the supply and accessibility of food [25]. Restrictions should be gradually relaxed as the pandemic spreads. These conclusions are consistent with the result of our research. The main research gap lies in the policy analysis of a single country or region. Food security policies usually involve multiple policy objectives, such as controlling the epidemic, stabilizing the food supply, restarting the economy, and social security, etc. However, there may be conflicts between these policy goals. For example, developing countries, due to limited resources that can be allocated, need to make trade-offs between blockade and control of the epidemic, food security, and economic development, so as to protect their own interests to the greatest extent. Compared with existing research, our research vividly demonstrates the panorama of food security policies in different types of countries, and the balance of different policy objectives at different stages. Countries need to adjust their policy objectives in a timely manner according to the domestic epidemic and food security situation to maximize the use of resources.

This article is a policy analysis. The lack of quantitative research on the impact of COVID-19 on global food security and post-assessment of countries’ response policies are limitations of this article. The data source of our research is the FAPDA database, which may not contain all countermeasures, and the policies of some countries need to be updated. In this article, four typical countries were selected for policy review and comparative analysis. Due to the degree of impact of the epidemic and the differences in national conditions, these four countries may not be representative of all countries, but they can still reflect the general situation and policy characteristics of similar countries. At present, the global spread of COVID-19 has been initially controlled. In addition to promulgating control policies, the effects of policies and the food security status of the people should also be closely monitored and need more quantitative research. In addition, research on policy comparison and experience sharing needs to be further strengthened in order to minimize the impact of the epidemic on food security.

### 4.2. Policy Implications

Public health emergencies such as COVID-19, due to their accidental and unpredictable nature, are generally not taken seriously at the beginning. However, the health risks, food security risks, and economic risks caused by them are widespread. The response policies implemented by different countries are essentially risk management measures. Based on the results of existing policy research, we hope to build a multi-level, rapid response global food security risk prevention and emergency control mechanism. It is of great significance for rapid and reasonable responses to similar public emergencies in the future, reducing food security risks and economic losses.

Risk management includes a series of actions, listing all possible risks, assessing their impact, and reducing or avoiding losses. It is often used in many fields such as finance, business management, machine system safety, accident prevention, and natural disasters [56]. In the face of uncertainties, strategies (decisions) can include three main types: precautionary actions (policy design, resource allocation, and water and food reserves), control actions in the process (social security, social distance, supply chain management, etc.), and adaptive adjustments (target adjustments, experience sharing) after observing and evaluating policy effects. To prevent and control COVID-19 and similar public health emergencies, preventive and adaptive strategies are adopted to make the prevention system sufficiently flexible and robust [56].

**Figure 7:**
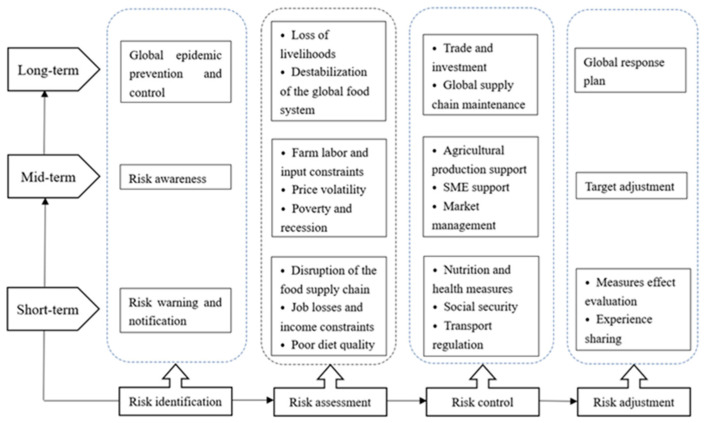
Food security risk response policy framework based on the perspective of risk management.

The current measures remain in the risk control during the event, and the risk response measures before and after the event are insufficient. In fact, an accurate and rapid risk identification mechanism can minimize the adverse effects of COVID-19 on food security. Objective risk assessment can provide a basis for government decision-making. In the process of risk control, appropriate risk adjustments based on changes in the epidemic can maximize the use of resources and reduce policy gaps and waste. Therefore, from the perspective of risk management, combining existing research progress, we try to build a policy framework for COVID-19 food security risk response from risk identification, risk assessment, risk control and risk adjustment, as shown in Figure 7, and propose policy implications.

From the perspective of risk identification, all countries still need to work together to improve risk awareness and risk identification capabilities, strengthen research and risk early warning of zoonosis, and control similar public emergencies from the source.

From the perspective of risk assessment, food security risks caused by COVID-19 have attracted the attention of many countries and international organizations. FAO is assessing the potential impact of COVID-19 on livelihoods, global food trade, food supply chains, etc., helping countries to foresee and alleviate the possible damage. Evaluating the impact of COVID-19 needs to be objective and reasonable. If the threat of COVID-19 is underestimated, it may lead to ineffective control. In contrast, it may cause panic and waste national resources.

From the perspective of risk control, policies are conducive to the short-term control of the pandemic while ensuring the availability of food, and are also important for restoring production and restarting the economy. COVID-19 not only affects people’s diets and lifestyles, but also has a profound impact on trade and investment, national relations, and geopolitical patterns. For countries that rely on food imports, developing regional economies is a good choice. For example, the African Continental Free Trade Agreement can unite African countries to reduce tariffs on medical supplies and agricultural products and regulate their domestic prices [28].

From the perspective of risk adjustment, in the short term, countries need to comprehensively evaluate the effects of their policies and make adjustments based on the trends of the pandemic and risk control objectives. Developed countries have the ability to take into account multiple risks, while less developed countries often have limited resources and capabilities. This requires governments to coordinate the relationship between humanitarian interventions and development interventions, and make necessary choices at different stages of the pandemic. In the long term, it is necessary for countries to strengthen policy communication, redesign the global food and agricultural governance system, and establish an effective coordination mechanism between global and local governance. The new global food governance system should strengthen the adaptability and anti-risk capabilities of the food system, coordinate and resolve policy conflicts between countries, and provide support for reasonable policy formulation.

## 5. Conclusions

COVID-19 has exacerbated the fragile food security situation globally, especially in developing countries. Ensuring access to food is a common challenge faced by all countries in the short term, but COVID-19 has further highlighted the uncertainty of long-term food supply in less developed countries. Different food security policies have implemented based on the national conditions. The response measures of developed countries represented by Italy are predominantly producer oriented, and more resources are used to support small- and medium-sized enterprises, focusing on the long-term recovery and development of the national economy affected by COVID-19. The response measures of less developed countries, represented by Malawi, are predominantly consumer oriented and trade oriented, paying more attention to how to control the pandemic in the short term while ensuring that people can get enough food. Food importing countries, represented by China, have adopted production support measures such as production subsidies and input distribution to stabilize domestic food market prices and strive to ensure national food supply. Food exporting countries, represented by Argentina, have gradually relaxed their trade policies. The initial import and export restrictions were to ensure a stable domestic food supply and control the spread of the epidemic, and then the agricultural product export promotion policy aimed to stimulate the country’s agricultural and economic recovery through exports. Food security policies have achieved good results, especially for ensuring domestic food availability and access in the short term. However, in the long run, in order to maintain the stability of the food system, countries still lack policy coordination and communication mechanisms. How a global food security community can be built is worthy of consideration.

## Figures and Tables

**Table 1:** Comparison of agricultural conditions in four countries.

Indicators	China	Italy	Malawi	Argentina
GDP per capita (current US dollars)	10,261.68	33,189.57	411.55	10,006.15
Cultivated land (hectare per capita)	0.09	0.11	0.22	0.90
Grain production (metric tons per capita)	0.44	0.27	0.20	1.70
Cereal imports/Cereal production (%)	4.38	86.49	5.05	0.02
Cereal exports/Cereal production (%)	0.44	8.63	0.08	55.19

Note: The data source is the statistical database of the World Bank and FAO.

**Table 2:** Main policy responses in the field of food security under COVID-19.

	Consumer Oriented	Producer Oriented	Trade Oriented
Africa	In-kind food transfer (10), Nutrition and health policy (9), Subsidies on fuel, power and water (8), Unspecified social protection measures (7), Unconditional cash transfer (7)	Institutional measure (17), Transport regulation and infrastructure (13), Public/mutual fund and contingent risk financing (11), Public institution (8), Access to credit (7)	Macroeconomic policy (8), Agricultural expenditure in the national budget (3), Other trade and trade-related measures (2), Export tax (1), Import tariff (1)
North America	Unconditional cash transfer (1), Production subsidies (1)	Financial support through public banks (2), Production subsidies (1), Support to productive assets (1), Risk management measures (1)	
Asia and the Pacific	Conditional cash transfer (13), Sanitation and hygiene (9), Transport regulation and infrastructure (8), Unspecified production support (8), Unconditional cash transfer (7)	Transport regulation and infrastructure (18), Financial support through public banks (12), Unspecified production support (12), Access to credit (9), Institutional measure (8), Government procurement through imports (8)	Macroeconomic policy (9), Agricultural expenditure in the national budget (7), Transport regulation and infrastructure (5), Export ban (5), Trade facilitation (4)
Europe	Unemployment compensation (6), Financial support through public banks (6), Unconditional cash transfer (6), Employment programs (6), Access to credit (6)	Financial support through public banks (13), Access to credit (11), Public/mutual fund and contingent risk financing (9), Production subsidies (7), Institutional measure (7)	Agricultural expenditure in the national budget (6), Export quota (2), Import ban (2), Import tariff (2), Macroeconomic policy (2)
Latin America and the Caribbean	Unconditional cash transfer (10), In-kind food transfer (6), Credit for consumption (5), Price control (5), School feeding (5)	Promotion of farmer markets or community markets (7), Transport regulation and infrastructure (6), Access to credit (4), Unspecified credit and finance facility (4), Financial support through public banks (4)	Macroeconomic policy (4), Export ban (1), Import quota (1), Import tariff (1), Other measures that affect imports (1)
Near East	Conditional cash transfer (7), Price control (6), Unconditional cash transfer (6), In-kind food transfer (5), Unemployment compensation (4)	Institutional measures (9), Transport regulation and infrastructure (8), Financial support through public banks (6), Access to credit (5), Public institution (5)	Export ban (4), Import tariff (3), Macroeconomic policy (3), Government procurement through imports (2), Trade facilitation (2)

Note: The data source is the FAPDA database of FAO. We listed the top 5 policies most adopted in each region. “()” refers to the number of countries that have adopted this measure, and the data is as of 31 December 2020.

**Table 3:** The threat of COVID-19 to food security and household income in Malawi.

Round	1	2	3	4	5	6	7	8	9	10	11
Food	67.1	63.5	63.7	-	65.5	62.9	62.8	62.7	54.9	-	40.0
Income	89.8	89.2	86.6	75.9	74.5	70.1	86.7	80.0	70.6	-	66.1

Note: The table shows the results of the 11 rounds of the High-Frequency Phone Survey on COVID-19 in Malawi, including the percentage of people who worry about not having enough food, and the percentage of people say that COVID-19 is a substantial threat to household income.

## Data Availability

Publicly available datasets were analyzed in this study. This data can be found here: [http://fapda.apps.fao.org/fapda/#main.html] (accessed on 10 January 2021).
